# Leans Illusion in Hexapod Simulator Facilitates Erroneous Responses to Artificial Horizon in Airline Pilots

**DOI:** 10.1177/0018720820975248

**Published:** 2020-12-03

**Authors:** Annemarie van den Hoed, Annemarie Landman, Dirk Van Baelen, Olaf Stroosma, M. M. (René) van Paassen, Eric L. Groen, Max Mulder

**Affiliations:** 1 2860 Delft University of Technology, The Netherlands; 2 312992 TNO Soesterberg, The Netherlands

**Keywords:** display, aviation, spatial disorientation, simulation, perception

## Abstract

**Objective:**

We tested whether a procedure in a hexapod simulator can cause incorrect assumptions of the bank angle (i.e., the “leans”) in airline pilots as well as incorrect interpretations of the attitude indicator (AI).

**Background:**

The effect of the leans on interpretation errors has previously been demonstrated in nonpilots. In-flight, incorrect assumptions can arise due to misleading roll cues (spatial disorientation).

**Method:**

Pilots (*n* = 18) performed 36 runs, in which they were asked to roll to wings level using only the AI. They received roll cues before the AI was shown, which matched with the AI bank angle direction in most runs, but which were toward the opposite direction in a leans-opposite condition (four runs). In a baseline condition (four runs), they received no roll cues. To test whether pilots responded to the AI, the AI sometimes showed wings level following roll cues in a leans-level condition (four runs).

**Results:**

Overall, pilots made significantly more errors in the leans-opposite (19.4%) compared to the baseline (6.9%) or leans-level condition (0.0%). There was a pronounced learning effect in the leans-opposite condition, as 38.9% of pilots made an error in the first exposure to this condition. Experience (i.e., flight hours) had no significant effects.

**Conclusion:**

The leans procedure was effective in inducing AI misinterpretations and control input errors in pilots.

**Application:**

The procedure can be used in spatial disorientation demonstrations. The results underline the importance of unambiguous displays that should be able to quickly correct incorrect assumptions due to spatial disorientation.

## Introduction

In modern aircraft, the main task of airline pilots is to monitor the automatic systems and intervene when the automation fails. Such interventions often require a prompt and correct interpretation of the instruments, which may be difficult after prolonged periods of eventless flight and distraction (Strauch, 2017). In an analysis of flight path management, vulnerabilities in pilot knowledge and skills were identified for manual flight operations (Federal Aviation Administration [FAA], 2013). Over 60% of investigated accidents involved manual handling errors, and these errors strongly co-occurred with transitions from automated control (FAA, 2013, p. 231).

In several recent accidents involving manual handling errors, confusion about the bank angle was implied. Examples include Kenya Airways flight KQA507 (Cameroon Civil Aviation Authority, 2010), Flash Air flight 604 (Bureau d’Enquêtes et d’Analyses pour la Sécurité de l’Aviation Civile, 2009), and Crossair flight 498 (Aircraft Accident Investigation Bureau, 2002). In these accidents, the pilot flying exerted a roll input *opposite* to the required direction, which is also referred to as a roll reversal error. It has been argued that these errors may result from an ambiguity of the artificial horizon, or attitude indicator (AI), which is the main display system to determine the aircraft bank angle (e.g., Johnson & Roscoe, 1972; Previc & Ercoline, 1999; Roscoe, 1968).

**Figure 1:**
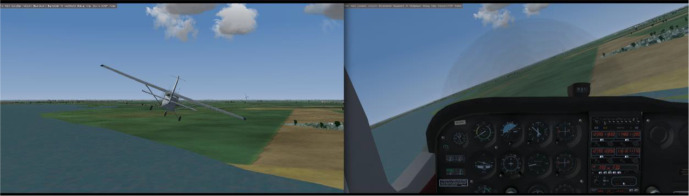
A situation of a bank angle from an external perspective, seen from behind the aircraft (left), and as seen from the cockpit (right).

The conventional inside-out or moving-horizon AI is designed so that the airplane symbol remains fixed on the display and the horizon rotates in the opposite direction of the control inputs. In other words, it is designed to mimic the outside view (“principle of pictorial realism”; Roscoe, 1968; see Figure 1). However, this principle may not be optimal for the AI, as displays inside the cockpit are thought to be processed by a different neurological system than the outside scenery (Previc & Ercoline, 1999). In the case of the AI, the “principle of the moving part” (Johnson & Roscoe, 1972) may be more important, which states that humans tend to control the part of a display that moves and perceive static elements as being the background. This principle is violated in the design of the moving horizon AI. Hence, in situations of high workload, surprise, or stress, pilots may revert to heuristics, and be inclined to control the moving horizon symbol *as if* it were the aircraft symbol.

In experiments intended to evaluate the AI, pilots were found to make 4.5%–8.7% roll reversal errors when rolling to level from previously unknown bank angles (Beringer et al., 1975; Müller et al., 2018). In-flight, pilots performed better with 1.5%–4.9% errors (Beringer et al., 1975; Hasbrook & Rasmussen, 1973; Roscoe & Williges, 1975). These outcomes show that there is indeed an ambiguity of the AI indicated bank direction, even for pilots. In these experiments, the effect of pilots’ assumptions regarding the bank angle was not tested. However, in the accident examples mentioned earlier, spatial disorientation seemed to have contributed to the fatal outcome. Spatial disorientation refers to having an erroneous sense of the aircraft attitude and motion relative to the earth, caused by misleading vestibular or other motion cues. Spatial disorientation has been estimated to have contributed to 12% of loss of control accidents, and to 24% of all fatalities in air carrier operations between 1996 and 2010 (Belcastro et al., 2017). An incorrect assumption of the bank angle is the most prevalent form of spatial disorientation in aviation, called the “leans” (Gillingham, 1992; Holmes et al., 2003; Pennings et al., 2020). It is a somatogyral illusion that can occur because the semicircular canals of our vestibular system do not sense low roll accelerations or sustained roll motions. A subthreshold roll acceleration may cause a pilot to assume that the aircraft is still flying level while it is actually banked. Then, when an unnoticed bank has developed, a *super*-threshold motion toward level flight may cause the pilot to incorrectly assume a bank angle toward the *opposite* side. Illusions like the leans are most likely to occur when outside visibility is low, forcing pilots to use their instruments for determining the aircraft attitude.

Recent studies have shown that an incorrect assumption of the bank angle increases the likelihood of making an RRE both in-flight as in a fixed base simulator (Landman et al., 2019; Landman et al., 2020). Error rates increased from 5%–9.8% when no bank angle was expected to 63%–75% when a bank angle was expected in the opposite direction. These experiments were, however, performed with nonpilots. Thus, the first objective of the current study was to investigate the effect of expectation, resulting from leans motion cues, on the occurrence of roll reversal errors in airline pilots. Pilots may not be as susceptible to these errors as nonpilots, as they are trained and experienced in ignoring misleading motion cues, as well as in reading the AI. A recent simulator study found that pilots indeed performed better than nonpilots in maintaining level flight while being exposed to leans motion cues, but this was without having the AI visible (Lewkowicz et al., 2020). A second objective was to develop a new procedure and motion profile for a hexapod simulator, in order to instill the leans illusion as accurately as possible. It was assumed that if pilots made interpretation and control errors corresponding with the motion cues, this would indicate that the procedure was effective in inducing the leans illusion. A third objective of the current study was to test whether more experienced pilots were less affected by the leans illusion than less experienced pilots.

## Method

### Participants

Eighteen airline pilots participated in the study. All were familiar with flying medium- to large-sized aircraft using a W-shaped control column or yoke (such as in Boeing aircraft), which was used in the experiment. Pilots were divided into two experience groups: low experience (*n* = 8) with less than 5000 flight hours, and high experience (*n* = 10) with more than 10,000 flight hours. Participant characteristics are displayed in Table 1. This research complied with the tenets of the Declaration of Helsinki and was approved by the human research ethics committee of the university. Informed consent was obtained from each participant.

**Table 1:** Characteristics of the Participants

	Group	
	Low Experience	High Experience
Gender: Female (*n*)	1	0
Gender: Male (*n*)	7	10
Rank: Captain (*n*)	0	9
Rank: First officer (*n*)	4	1
Rank: Second officer* (*n*)	4	0
Flight hours (hr)	2300, *SD* = 1930	16,989, *SD* = 3213
Years employed (yrs)	4.0, *SD* = 3.7	30.4, *SD* = 5.6
Age (yrs)	28.8, *SD* = 7.1	55.3, *SD* = 6.4
Sleep in previous two nights (hr)	14.0, *SD* = 2.0	14.6, *SD* = 2.0

*Note*. *The rank indicates a novice pilot who is third in line of command on long haul flights.

### Apparatus

The experiment was performed in the Simona Research Simulator (SRS) at the faculty of Aerospace Engineering of the Delft University of Technology (see, Stroosma et al., 2003). The SRS is a six degrees-of-freedom full-motion simulator with a hydraulic hexapod motion system, which can realize accelerations below human vestibular perception (see Heerspink et al., 2005). The pilot was seated in the left-hand seat of the cockpit, which featured a collimated 180° horizontal × 40° vertical field of view screen. The images were rendered by FlightGear software and projected with the use of high-resolution computer-generated images using three Digital Light Processing (DLP) projectors.

**Figure 2:**
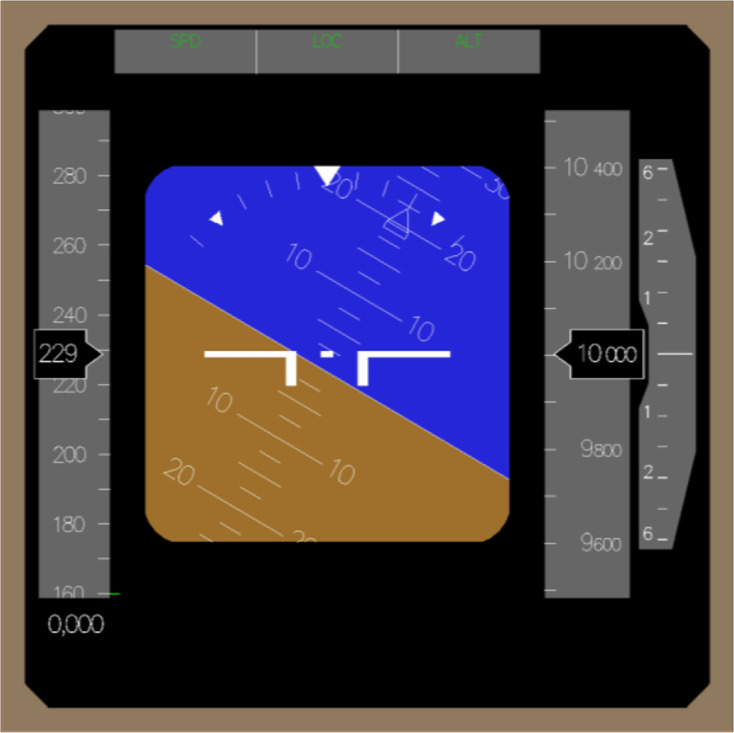
The primary flight display that was as used in the experiment, with the attitude indicator indicating a 30° bank to the left.

The simulation used an aircraft flight dynamics model representative of a medium-size twinjet aircraft (Airbus A320). Participants were only able to control the roll axis using a control-loaded column. The only display that was provided was a simplified digital primary flight display (PFD; see Figure 2), showing the AI, airspeed, vertical speed, altitude, and autopilot status. Audio simulation featured a constant engine and wind noise, and the autopilot disconnect alert. Pilots wore noise-canceling headphones to prevent them from hearing the simulator motion system, but they could hear the autopilot disconnect alarm. A 10-inch tablet was used for the secondary (distraction) task.

### Briefing and Familiarization

The tasks were performed as single-pilot crews using the left-hand seat of the simulator. Pilots were tested either in the morning (9:00–12:00 AM) or in the afternoon (1:00–4:00 PM). Each pilot was first briefed, provided informed consent, and filled in a general questionnaire. Pilots were told that the experiment was about “assuming manual control after a period of automatic flight and distraction.” Following a period of automatic flight, they were to take the control column when the autopilot off alert sounded, wait for the AI to appear, and then roll the aircraft level using the AI. They were instructed to respond *immediately* when the AI appeared, as “an intuitive response was desired and reaction time would be one of the outcome measures.” Pilots familiarized themselves with the simulator and controls by flying a number of turns for approximately 3 min.

### Stimuli

**Figure 3:**
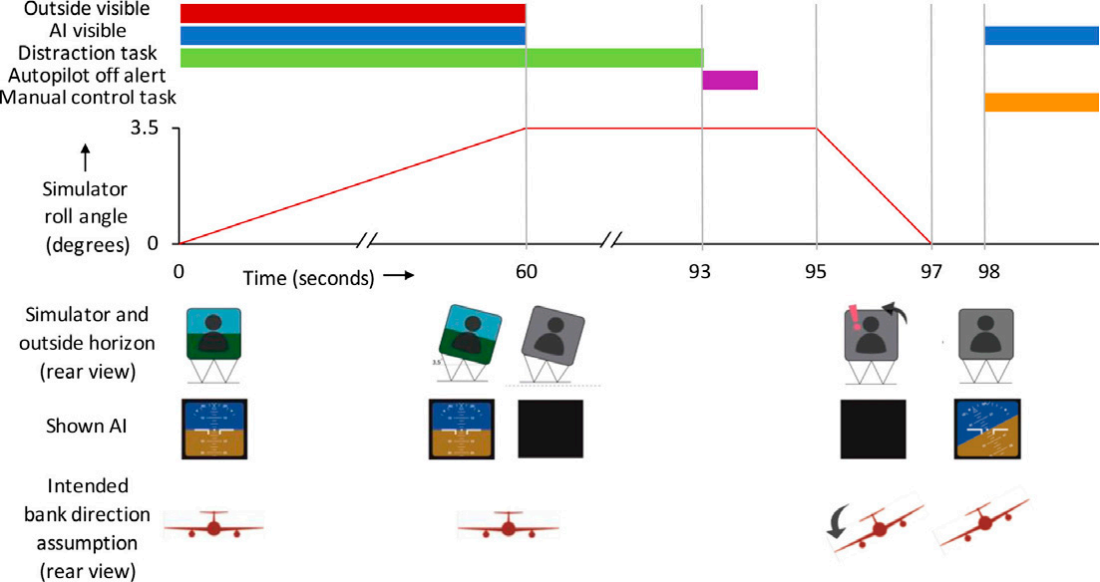
A timeline of the stimuli in a run. This example shows a run in the leans-opposite condition. AI = attitude indicator.

A leans-inducing motion profile was developed and tuned with nine nonpilots (see Landman et al., 2021, for a full description of this pilot study). Advantages of this motion profile in comparison to existing leans motion profiles (see Bles, 2008) are as follows: (1) Cues that would not occur in an in-flight leans illusion (i.e., pitch or yaw cues, or a pronounced roll angle) are minimized. (2) The profile can be implemented in a hexapod simulator. (3) The simulator platform is upright and steady when the pilot performs the response task. The latter is important to rule out the possibility that pilots are responding to simultaneously occurring motion cues (e.g., as a postural reflex) instead of to the leans illusion. An overview of the stimuli and the intended sensation is displayed in Figure 3.

During the entire procedure, the speed remained fixed at approximately 230 knots, and the altitude at 10,000 feet. There was also a continuous light turbulence (using a Dryden model of turbulence with σ = 1.0, *L* = 2000, *V* = 200; Liepmann, 1952) added to the vertical axis of the simulator throughout the whole experiment, to mask motion onsets.

The procedure started with straight and level flight with the autopilot engaged. The pilots performed a distraction task, which was a version of the Multi Attribute Task Battery (MATB-II; Santiago-Espada et al., 2011). The MATB-II was performed on a tablet attached to the surface of the center pedestal to the pilots’ right-hand side, which required the pilot to slightly lean and be turned to the side. The tracking task of the MATB-II was not included.

After approximately 10 s of flight, the motion profile started with a prepositioning phase (Figure 3), during which the simulator platform was slowly (in 60 s) tilted to a roll angle of 3.5° (.06 radian [rad]), while the AI and the outside view continued to indicate level flight. A maximum roll acceleration of 1.696 × 10^-5^ rad/s^2^ and a maximum angular velocity of .001 rad/s were used for prepositioning, both of which are under the human perception thresholds of .0349 rad/s^2^ and .002 rad/s, respectively (Gundry, 1977; Heerspink et al., 2005).

After the prepositioning phase, a situation was simulated of the pilot being momentarily distracted from the instruments and from the outside view. The AI and the outside vision were covered (turned black) for the next 33 s, while the platform maintained a steady roll angle of 3.5° (Figure 3). This adaptation phase was included to induce vestibular adaptation to the roll angle. According to earlier studies (e.g., Crane, 2012), prolonged exposure to a roll angle can cause an aftereffect, wherein a subsequent roll angle toward the opposite direction is overestimated in that direction. The adaptation phase was also required to realistically simulate a situation in which the aircraft could roll below the pilots’ perceptual threshold to a new bank angle.

At the end of the adaptation phase, the autopilot disconnect alert sounded, upon which the pilot was to prepare for intervention by facing the still-covered AI and by holding the control column. Two seconds after the alert, the simulator platform was tilted back to level in 2 s, with a maximum roll rate of .03 rad/s and roll acceleration of .075 rad/s^2^. This was *above* the documented perceptual threshold of the vestibular system (Gundry, 1977; Heerspink et al., 2005), while it also presented tactile cues as the motion shifted the pilot in the seat. One second after this super-threshold roll cue ended, the AI was shown again. The pilot would then use the AI to immediately roll to wings level.

### Conditions

The procedure described above was repeated in a number of runs, with each run featuring one of the following variations of the procedure:

1. Filler runs. The AI shown after the motion cues was banked 30° in the same direction as the super-threshold roll cue. The AI shown at 98 s in Figure 3 would thus be banked to the left. This variation was used to have the pilots gain trust in the motion cues. In a second variation of these filler runs, the platform remained steady and upright throughout the whole procedure and the AI shown at the end was presented wings level. This variation was included to reduce the possibility of pilots being surprised by not having to give an input in the leans-level condition described below.
2. Baseline condition runs. In this variation, the platform remained steady and upright throughout the procedure, while the AI was shown at the end with bank angle of 30° (left or right). This simulated an in-flight situation of a subthreshold roll to a bank angle. The error rate was expected to be around 4.5%–8.7% based on studies in fixed-base simulators (Beringer et al., 1975; Müller et al., 2018).
3. Leans-opposite condition runs. This variation is shown in Figure 3. The AI shown after the motion cues is banked 30° in the opposite direction of the super-threshold roll cue. Here, an in-flight situation was simulated of a subthreshold roll to a bank angle of 33.5°, followed by a super-threshold rollback to 30°.
4. Leans-level condition runs. This variation featured the same leans cues as the baseline and leans-opposite runs, but the AI at the end (Figure 3; 98 s) presented wings-level flight. This simulated an in-flight situation of a subthreshold roll to a bank angle of 3.5°, and then a super-threshold rollback to level. This condition was included to test whether pilots made spontaneous errors that were based directly on the leans cues, while neglecting the AI.

The pilots performed the runs in two sessions of continuous flight, which lasted 40 min each. There was a 10-min break between the sessions. The first session started with eight filler runs, of which the first two runs were practice runs.The second session started with six filler runs. In the subsequent 12 runs of each session, there were six more filler runs and two runs of each of the test conditions (i.e., baseline, leans-opposite, and leans-level). One “level” filler run (i.e., no motion and wings-level AI) was featured in the six filler runs at the start, and one in the next six filler runs of the session.

The test conditions and filler runs all featured an equal number of runs with the fast roll cue toward the left and right. Several sequences of the last 12 runs in each session were created using the Latin Square method, so that none of the test condition runs were systematically preceded by a certain type of run. Each session in all sequences ended with a test condition run, and the sequences were counterbalanced between the experience groups. Table 2 shows an overview of the different run types.

**Table 2:** Overview of the Runs Used in the Experiment

Type of Run	Roll Cue	AI Bank Angle	AI Bank Direction	*N* Runs in Total	*N* Runs per Session
Filler with motion	Yes	30°	Matching roll cue	22	10
Filler without motion (level)	No	0°	-	4	2
Baseline *	No	30°	Left or right	4	2
Leans-opposite *	Yes	30°	Opposite to roll cue	4	2
Leans-level *	Yes	0°	-	4	2

*Note*. * Test conditions. AI = attitude indicator

### Dependent Measures

The following variables were obtained in the test conditions.

#### Errors

An error was defined as a roll input away from level following AI presentation, which caused the control column to exceed 1° of roll deflection (compare Lewkowicz et al., 2020).

#### Error severity

When an error was detected, the maximum bank angle deviation toward the wrong direction was measured. The initial aircraft bank angle when starting the response was subtracted.

#### Reaction time

This was the time between AI presentation and the first input (i.e., column deflection rate >0 rad/s). This was measured for correct inputs only. If there was an initial incorrect interpretation before a correct response, there may be a moment of hesitation and starting the correct response may thus require more time.

#### Subjective workload of the distraction task

As a check that the distraction task induced sufficiently high workload, the subjective workload of this task was rated on the NASA Task Load Index (NASA-TLX; Hart & Staveland, 1988) after each session was completed. In this scale, workload is rated separately for mental demand, physical demand, temporal demand, performance, effort, and frustration.

### Hypotheses

More errors, more severe errors, and longer reaction times were expected in the leans-opposite condition than in the baseline condition, due to the leans protocol. More errors and more severe errors were expected in the leans-opposite condition compared to the leans-level condition, due to the possibility of misinterpreting the AI (i.e., experiencing a horizon control reversal) in the leans-opposite condition. High experience was expected to lead to fewer and less severe errors, as well as shorter reaction times, especially in the leans-opposite condition due to fewer misinterpretations. No differences were expected in the workload ratings of the distraction task.

### Data Analysis

Generalized Estimating Equations (GEE) tests were performed for ordinal data (error percentage), with the factors: Group (low and high experience), Condition (baseline, leans-level, and leans-opposite), and the Group × Condition interaction. For linear data (error severity and reaction time), a mixed model (Group × Condition) analysis of variance (ANOVA) was performed. As reaction time was limited to correct responses, the leans-level condition was not included in this analysis.

Post-hoc comparisons between conditions were performed with Wilcoxon signed rank (for ordinal data) or paired-samples *t*-tests (for linear data). Two comparisons were performed: between the leans-opposite and baseline condition, and between the leans-opposite and leans-level condition. We corrected for two comparisons using Bonferroni correction (required *p* = .025).

To test whether there was a learning effect, the error frequency in the whole group in the first run of each condition was compared with that in the last run of each condition using a McNemar test.

Workload ratings were compared between the groups using independent-samples *t*-tests.

## Results

None of the pilots reported any motion sickness issues when asked halfway into the experiment and at the end. Table 3 shows an overview of the performance outcomes.

**Table 3:** Overview of the Performance Outcomes

	Condition	Low Experience		High Experience		Total	*N*
		Mean (*SD*)	*N*	Mean (*SD*)	*N*	Mean (*SD*)	
Errors (%)	Baseline	6.3 (11.6)	8	7.5 (16.9)	10	6.9 (14.4)	18
	Leans-opposite	18.8 (17.7)	8	20.0 (19.7)	10	19.4 (18.3)	18
	Leans-level	0.0 (0.0)	8	0.0 (0.0)	10	0.0 (0.0)	18
Error severity (degrees)	Baseline	2.6 (2.5)	2	3.9 (.1)	2	3.2 (1.6)	4
	Leans-opposite	3.0 (2.3)	5	4.4 (2.1)	6	3.8 (2.2)	11
	Leans-level	-	-	-	-	-	-
Reaction time (s)	Baseline	1.71 (.34)	8	2.15 (.29)	10	1.95 (.38)	18
	Leans-opposite	1.66 (.23)	8	2.15 (.45)	10	1.93 (.44)	18
	Leans-level	-	-	-	-	-	-

### Errors

The GEE analysis of the error percentage showed that there was a significant main effect for Condition, χ^2^ = 9.16, *p* = .002, but not for Group, and there was no significant Condition × Group interaction. The post-hoc comparisons showed that there was a significant difference between the leans-opposite (median = 25%) and baseline condition (median = 0%), *Z* = −2.71, *p* = .007, and between the leans-opposite and leans-level condition (median = 0%), *Z* = −3.07, *p* = .002. An interesting finding was that pilots also made 3.9% errors in the filler runs, in which the direction of the fast roll cue matched with the shown AI.

The McNemar test showed that there was a significant learning effect in the leans-opposite condition. Seven pilots, or 38.9%, made an error in the first run, whereas none of the pilots made an error in the final run, *p* = .016. The error percentage decreased linearly over the four runs. In the baseline condition, two pilots (11.1%) made an error in the first run and none in the last run, but this difference did not reach significance. No errors were made in the leans-level condition.

### Error Severity

**Figure 4:**
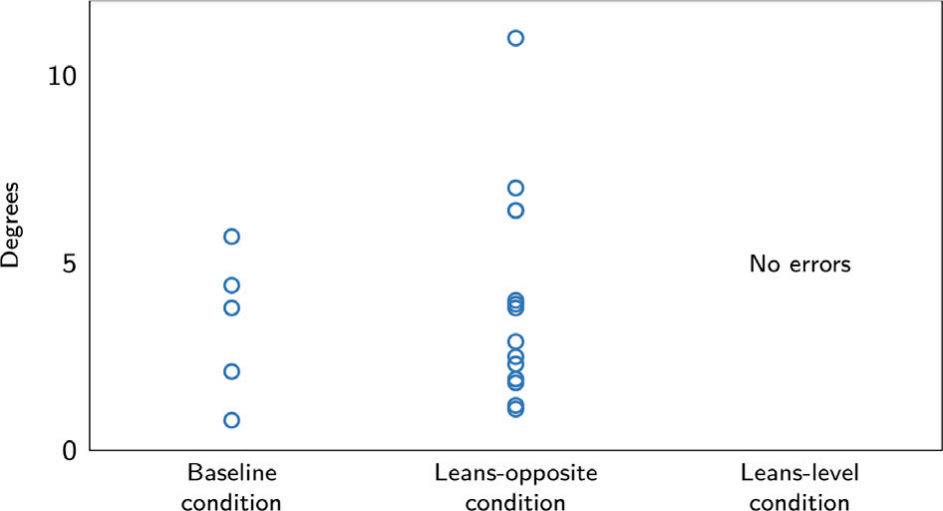
The error severity, in degrees exceeding of 30° bank, of all detected errors.

All errors are displayed in Figure 4. The severity of the errors was, as expected, highest in the leans-opposite condition (Figure 4 and Table 3); however, no statistical test could be performed due to an insufficient number of pilots making an error in both conditions.

### Reaction Time

On average, pilots responded around 2 s following the AI presentation. There was no significant main effect of Condition, no significant Condition × Group interaction, but there was a significant effect of Group, *F*(1,16) = 9.20, *p* = .008. The high experienced group responded on average .46 s slower than the low experience group, which was in contrast to our hypothesis.

### Subjective Workload of the Distraction Task

Pilots rated the mental demand of the secondary task at 62.4 points (Table 4), which is around the midpoint of the scale (i.e., 50). The high experience group gave higher ratings to physical demand, *t*(1,16) = 2.19, *p* = .043, and frustration *t*(1,16) = 2.09, *p* = .043, than the low experience group.

**Table 4:** NASA-TLX Workload Ratings of the Secondary MATB-II Task. Scores Are Averaged Over the Two Sessions

	NASA-TLX score Mean (*SD*)
Mental demand (0–100)	62.4 (14.5)
Physical demand (0–100)	36.5 (15.5)
Temporal demand (0–100)	45.1 (15.0)
Performance (0–100)	39.3 (17.2)
Effort (0–100)	59.3 (16.6)
Frustration (0–100)	28.9 (23.3)

*Note.* MATB-II = Multi Attribute Task Battery; NASA-TLX = NASA Task Load Index

## Discussion

We presented pilots with a leans protocol, which featured a roll motion cue in the opposite bank direction of a subsequently shown AI. We simulated an in-flight situation of a subthreshold roll to 33.5° bank, and a super-threshold rollback to 30°. In response to these cues, 38.9% of pilots made a roll reversal error during the first encounter, and 19.4% errors were made on average in the four runs. This error rate was significantly higher than in the baseline condition (6.9%), which featured no roll motion cues followed by a banked AI. This indicates that the errors were indeed caused by the leans protocol. The error rate was also significantly higher than in the leans-level condition (0.0%), which featured the same roll motion cue but followed by a wings-level AI. This indicates that the leans cues induced an interpretation error of the AI, and that pilots were not responding based only on what they felt. In line with previous experiments (Landman et al., 2019; Landman et al., 2020), this points towards an inherent ambiguity in the moving-horizon AI bank angle indication.

The error rate found in this study for airline pilots is lower than that in nonpilots, whose expectation of bank angle was manipulated in a fixed-base simulator (75% error rate; Landman et al., 2020), or by true leans cues in-flight (58% error rate; Landman et al., 2019). This is to be expected, considering the pilot’s experience with both leans cues and reading the AI. Interestingly, the error rate in the leans-level condition was especially much lower in our pilots (0.0%) than it was in nonpilots in-flight (63%; Landman et al., 2019), indicating that pilots are more inclined to base their response on the instrument instead of on the roll motion cues. The error rate we found in our baseline condition without motion (6.9%) is similar to that in comparable conditions in other fixed-base simulator experiments with pilots (5.1%–8.7%; Beringer et al., 1975; Müller et al., 2018). Our experiment shows that the error rate in the baseline condition, even though it is already high from a safety perspective, is likely an underestimation of pilots’ difficulties with responding quickly to the AI when spatially disoriented.

When interpreting the error rates, it is important to note that we asked pilots to respond immediately, forcing them to make an intuitive response. This was done in order to simulate a response under high workload and stress, which is difficult to recreate controllably in a simulated setting. The errors we found were quickly corrected and did not exacerbate into dangerous situations. Despite our instruction, pilots responded somewhat slowly (ca. 2.0 s reaction time) compared to nonpilots in a comparable experiment (ca. .5 s; Landman et al., 2020). Perhaps pilots are more inclined to respond slower as this would be a more realistic response when in flight.

We found no differences in susceptibility to errors between pilots with less than 5000 flight hours and those with over 10,000 flight hours. It seems therefore that flying experience did not protect against these errors. However, the high experience group responded significantly slower than the low experience group, which is possibly the result of the age difference between the groups (Table 1; Fozard et al., 1994).

In contrast to nonpilots (Landman et al., 2020), there was a strong learning effect in pilots, as none of the pilots made an error in the fourth run. This is very promising for the use of hexapod simulators for spatial disorientation awareness training, as it suggests that pilots can very well suppress the effects of the misleading roll cues on their responses. The long-term retention of this learning effect is, however, not clear.

The results indicate that, even within the limitations of a hexapod simulator, the leans can be induced without additional unrealistic cues, to such an extent that it leads to erroneous inputs even in experienced pilots who have the AI as reference. For maximum effect, we made use of the effects of visual dominance, adaptation to a static roll angle, distraction, and prepositioning of the simulator. The advantages compared to other motion profiles are that the simulator platform is upright and steady following the cue, and that it features roll cues only. One pilot indicated that he had recently experienced the leans, and that the sensation in the simulator was highly similar. Some pilots did not consciously notice the super-threshold roll motion cues, but they still responded in line with the hypotheses. The developed leans procedure can be used to test the effect of spatial disorientation on the interpretation of displays with different bank indications (e.g., Beringer et al., 1975; Ewbank et al., 2016). However, it is important to note that the simulation has not been validated yet by comparing it with real flight, and that error rates may differ.

In conclusion, the current experiment shows that the bank angle direction on the moving-horizon AI was more often misinterpreted when professional pilots expected an opposite bank direction due to the leans protocol we developed. The study presents an effective hexapod simulator leans procedure that can be used in spatial disorientation awareness training, so that pilots may experience the illusion and its confusing effects in a safe setting. This leans procedure can be integrated in a more complex and realistic flight scenarios.

## Key Points

- The effect of pilot expectation due to spatial disorientation (leans) cues on interpretation of the attitude indicator was investigated.
- For this, a new leans procedure and simulator motion profile was developed for a hexapod simulator.
- The disorienting motion cues caused an increase in roll reversal errors by a factor of almost 3.
- The results show that incorrect expectations can have hazardous effects on display interpretation, and underline the importance of intuitive display design.
- The leans procedure, which we found to be effective, can be used for spatial disorientation research and training in a cost-effective hexapod motion simulator.
